# Delirium Tremens

**Published:** 1834

**Authors:** 


### REVIEWS AND BIBLIOGRAPHICAL NOTICES.

### Art. IV.—Delirium Tremens.

*Prize Dissertation* (*for* 1831,) *on* Delirium Tremens. By James Conquest Cross, M. D. *of Lexington, Kentucky.*

(*Transactions of the New-York State Medical Society, Vol. I.*)

According to the researches of Dr. Cross, people have continued to get drunk from the earliest ages of the world; but Delirium Tremens was not described till the latter part of the last century. Why it was not, our author is unable to find out. Perhaps it was confounded, in most cases, with drunkenness. Perhaps the more general cultivation of mind, in modern times, may predispose to this malady. If it really is, as seems probable, a more frequent disease in this century than before, it would be interesting, in a philosophical point of view, to discover the cause, though we are not aware of any special advantage, that would flow to society, from the discovery. If Delirium Tremens could be prevented by any other means than temperance, what would be the advantage? Would not the chapter of desolations from strong drink be still dark enough, if that item were stricken out? It would signify nothing, to prevent this or that evil consequence of drunkenness; such mitigation of its effects would only perpetuate the practice of drinking. Nearly in the same way, we view all the nostrums for the cure of drunkenness. As far as faith is reposed in them, they manifestly invite to intemperance. He who believes, that should he come to drink to excess, he can use a medicine that will enable him to "hold up," must *cœteris paribus*, be more likely to indulge himself, than if he saw no remedy ahead.

Had the respectable society of the Empire State, offered a prize for the best dissertation on the means of preventing drunkenness, instead of the cure of one of its occasional effects, they would, we beg leave to say, have acted with greater wisdom, though not perhaps under a more benevolent impulse.

We know of no case to which the good old adage, *an ounce of prevention is worth a pound of cure*, has such strict applicability, as that of drunkenness. We do not affirm—once a drunkard always a drunkard; but it cannot be predicted of any drunkard, that he will ever become a sober man; on the contrary, all the chances are against him; and as to him who has drunk until he induced Delirium Tremens, we are prepared to say from the observations of many years, that no one need hope to see him permanently restored. Now and then such an one makes a good resolution, and begins the work of reform; but ere long, relapses, *as the dog returns to his vomit,—as the sow that was washed, to wallowing in the mire.*

In reference then, to Delirium Tremens, and the other diseases which spring from intemperance, let us look rather to the *prophylacticks*, than the *methodus medendi*. Should nothing be done in this species of delirium, our patients, we incline to believe, would not much oftener die, than when the most approved plans of treatment are adopted. In a majority of cases, the encephalic irritation would spontaneously subside, and the patient recover—provided his constitution was not laid waste to an irrecoverable degree, by the previous habit.

Nevertheless, something remedial is called for, and we shall therefore proceed to analyze our author's dissertation. He first treats of the literary history of Delirium Tremens, and informs us that the earliest notice he can find of it, is a tract by Dr. S. B. Pearson, of England, dated in 1801. He next treats of its name, and gives us the following synomimes: *Brain fever*, *Mania a Potu*, *Mania e temulentia*, *Febris temulentia*, *Ænomania*, *Delirium ebriositatis*, *Encephalitis tremefaciens*, *Delirium Tremens*, the last of which he prefers. The Delirium Traumaticum of Dupuytren, he supposes to be nearly of the same character, with Delirium Tremens.

He next enquires, whether intoxication is necessary to the production of this malady, and concludes that it is not, which we think a correct opinion. We likewise concur with him in his next conclusion, that it is not indispensably necessary to the production of this disease, that the use of ardent spirits should be suspended. We admit that it has often seemed to follow such a suspension as a consequence, but in many of these cases, the patient no doubt, ceased to drink, because the delirium was forming.

Under the head of general character of the disease, our author has given a full, and, on the main, an accurate and graphical portraiture of the disease, which we shall transfer nearly entire, to our pages.

"In at least eight-tenths of the cases of this disease, it will be found to make its onset during that state of depression, which invariably supervenes upon the sudden relinquishment of the protracted and immoderate use of ardent spirits. Among the first evidences of the approach of delirium tremens, may be mentioned as the most general, a universal *malaise*, lassitude, coldness of the hands and feet, indistinct *horripilatio*, sometimes almost imperceptible, and uniformly imperfect.—There is frequently a considerable reduction, and sometimes a marked augmentation of heat of skin. The body generally, but particularly the face, is bedewed with an abundant and disagreeable cold, clammy perspiration. Sometimes the skin is hot and dry, and it frequently happens, that when perspiring, the fluid exhaled is comfortably warm. The pulse for the most part is remarkable for its slowness and feebleness; not yielding, in some cases, more than sixty-five beats in the minute. In some of the forms of this disease, it presents a character altogether different. Sometimes we find the pulse remarkably small, thready, quick, and so frequent, as to give more than 120 pulsations in the minute; sometimes gaseous, full, round, voluminous, but compressible; somestimes tense, resisting, and with difficulty compressed. The appetite is always impaired, frequently annihilated, and the patient generally experiences an insurmountable loathing for animal food. The tongue is usually moist, though sometimes dry. It presents numerous morbid appearances, and is sometimes perfectly clean. There is in some cases white fur, sometimes a sticky, glutinous slime, frequently white, and often of a yellow bilious hue. There is generally nausea, frequently vomiting, though in some cases the stomach is perfectly tranquil throughout the disease. Oppression and precordial anxiety are rarely ever absent. Frequently there are epigastric tumefaction and heat. There is sometimes an exception, but in a majority of instances, the head suffers from pain. This exists from simple headache to the most intense and insufferable torture. The spirits are generally gloomy and depressed, though sometimes in a high state of exhiliration. The expression of countenance is usually dejected and melancholy, though sometimes haughty, fierce and daring. Pervigilium commonly exists from the inception, and obstinately persists, until the disease is subsiding. If the patient should sleep, his naps are short, unrefreshing, and frequently interrupted by alarming and frightful dreams. The hands generally tremble: often the tongue, and sometimes the muscles of the extremities, are spasmodically affected. There are cases, however, in which there is no tremor; the hands exhibiting astonishing normal steadiness. The bowels are almost uniformly in an obstinate state of constipation. In some rare instances, they are found in a state of colliquative solubility.

The length of time during which the system labours under the symptoms just enumerated, before delirium is developed, is various, and cannot be determined with much precision. It depends upon a number of collateral circumstances, hereafter to be particularly mentioned. Generally, however, they will be found to exist from twenty-four to forty-eight hours, although its development will take place earlier in a number of instances.

The form when it is revealed is peculiar, and so far characteristic, as to be seldom developed in any other abnormal state of the system. Its conspicuous attribute consists in a belief of the existence of frightful objects, in positions in which, as Dr. Pearson has well remarked, it is physically impossible for them to be situated. The patient fancies that Satan is attached to the ceiling, and as his disease increases in intensity, his majesty appears to hover over him, and he will shrink as if there were reality in the impending danger. If he is a soldier, and has ever been in battle, the contortions of his body show that he is attempting to avoid the thrust of bayonets, that are on the point of being plunged into his vitals. His apartment is frequently crowded with his imaginary intruders. He is constantly pursued by an enemy, whose machinations it is his exclusive study to defeat or to elude. He is in great dread of assassination. The co-operation of his attendants is frequently solicited to assist him in defending himself against enemies, who wish to shoot or poison him, or to drive from his presence disgusting objects, that annoy him exceedingly, such as snakes, lice, rats, frogs, and vermin of every description, with which he imagines the floor of his chamber or his bed to be covered. Sometimes snakes are in his bed, attacking him at all points, which, from his deportment, seem to inflict real pain. It often happens that his apartment is so crowded with visitors, who are so zealous and indefatigable in their attendance upon him, that he complains of their keeping him from his regular hour of repose; sometimes they disturb him by repairing or altering the house; sometimes they severely castigate him with the lash; they pull off the bed clothes, dance, box, and go through various gymnastic exercises.

His loquacity is unintermitting; he holds colloquial intercourse with ideal personages. Imaginary sounds distress him exceedingly. A soldier cannot be persuaded that he is not arraigned for the commission of some crime, which is to subject him to the penalties of the military code, and his mind is altogether engrossed in preparing his defence. If religion has ever been a subject of deep and engrossing reflection, he supplicates for mercy. If he is subjected to personal restraint, the dilapidating condition of his pecuniary affairs imperiously demands his immediate attention. His confinement he is apt to suppose illegal, and designed to answer some individual or selfish purpose. Often, under the pressure of urgent business, he imagines he has walked or rode several miles, when he has not left his chamber. Under the impression that the house is about falling, and will crush him to death, he is exhausted in attempting to support its sinking walls. He is rarely satisfied with the situation of the furniture, particularly with that of his bed, which he will leave, if not prevented.

Solitude is frequently a state of dreadful agony. He entreats and conjures most pathetically not to be left alone, exposed to the unrelenting cruelty of the demons and evil spirits by which his room is crowded, and who only wait until he is unprotected to accomplish his extermination. No excuse, no apology, is sufficient to persuade him to suffer his attendants to retire. No reasoning or assurance can convince him that his apprehensions are unfounded.

Under this morbid excitement, he conceives plans, which it is perfectly impossible to execute; and often he is intent upon the execution of plans previously formed. Sometimes it has been observed, that individuals laboring under this species of delirium, have given proofs of a much richer, more exuberant and brilliant imagination, than when the intellectual faculties were in a state of perfect equilibrium. Occasionally they discover a grandeur of conception, pregnancy of fancy, and an eccentricity of thought, that fill the mind, inclined to the elegant pursuits of philosophy and literature, with unlooked-for pleasure.

To divert the mind from the subject of its delirious wandering, is frequently absolutely impracticable, and attempts to accomplish it often result in an aggravation of the symptoms. Injudicious opposition is apt to transport them with rage. Those that do not possess full belief in their absurdities, are treated with ineffable contempt. Deportment of a soothing and conciliating character may sometimes succeed in securing their confidence, but advantage should not be taken of the treacherous calm which results from it, to propound numerous or impertinent questions. This is almost certain to occasion confusion of thought, and aggravation of the delirium. It is a matter of astonishment to observe with what precision the expression of countenance corresponds with the emotions which disturb the mind, and the ideas that float wildly through the desolated mansion of thought.

Frequently patients display achievements of astonishing corporeal vigor, but it is generally of short duration, and is invariably succeeded by an almost overwhelming muscular feebleness.

It has been asserted, that this delirium always relates to and is exclusively engrossed with the patient's private affairs. This is doubtless very commonly the case; but when it is asserted to be universal, we dissent, from a conviction that it is unsustained by experience. It sometimes happens that he cannot be convinced that he is in his own house; and the calamity which he dreads is not always personal. The misfortunes of his friends constitute, on some occasion, the ground of his complaints.

At night, or if the apartment of the patient should be darkened, this delirium will certainly be exacerbated.

The symptoms above enumerated, may continue as long as ten days, but at the expiration of that time, convalescence must be established, or they will assume a character decidedly adynamic. Either of these events may, however, and actually do, in a majority of cases, supervene sooner, but ten days is the period beyond which a patient cannot labor under delirium tremens, except the symptoms be adynamic.

When, therefore, the disease has not been completely subjugated within the time designated, the following symptoms will be found to exist. Perspiration now issues almost in a stream, from every pore of the skin; the whole body trembles, and the hands in particular are almost paralytic. The pulse is now smaller, more tremulous, thready, and so exceedingly rapid, that in some cases, it is impossible to count it. The tremor of the hands, and subsultus tendinum, may, however, deceive us in regard to the strength of the heart. Under these circumstances, there is some difficulty in feeling the pulse, and if we are not careful, we may believe it to be so frequent as not to be numbered, and so feeble as to be almost imperceptible, when in fact, if the patient should become tranquil, as sometimes happens, we may find it not very frequent, nor very feeble. The tongue is dry, occasionly cracked, and encrusted; the countenance is suffused, and its expression anxious; the pupils are contracted; if he is able, the patient picks at the bed clothes, his delirium is now low, and muttering; the urine and excrement are passed unconciously. Sometimes he becomes insensible, and expires in a comatose, or apoplectic state."----pp. 22 27

Dr. Cross, in the next division of his subject, proceeds to inform us, that "there are no less than four separate and distinct varieties of Delirium Tremens. First, sthenic,—second, hypersthenic,—third, asthenic,—fourth, bilious. We do not, however, attach very great importance to these distinctions. That which is hypersthenic, may, in three hours, be made asthenic, by the use of the lancet, and to a complication with deranged biliary action, each of the first three varieties may be considered equally liable.

Passing over our author's remarks on the discrepancies between Dr. Coates and himself, in regard to the symptoms of the disease; and also, his views of its progress and diagnosis, as not presenting much of interest or novelty, we come to his digest of morbid anatomy, beginning with that of the Brain.

"First. That there is congestion of the veins of the brain, which we deduce from the testimony of Professor Chapman, Mr. Howship, Drs. Barkhausen, Shirlitz, Brown and Peace.

Secondly. Drs. Barkhausen and Snowden testify to the existence of a florid or reddish appearance of the brain.

Thirdly. That there is actual inflammation of the brain, is proved by the evidence of Professor Chapman, Drs. Barkhausen, Shirlitz, Howship and Hunt.

Fourthly. That inflammation is not of uniform occurrence, is established by the silence of Drs. Snowden, Brown, Sutleffe, Peace, and the positive declaration of Drs. Johnson and Barkhausen.

Fifthly. That there is occasionally an exudation of congulable lymph, is testified to by Drs. Blake and Hunt.

Sixthly. That sanguineous extravasation occurs, is proved by Professor Chapman and Dr. Peace.

Seventhly. That serous effusion beneath the arachnoid membrane, and into the lateral ventricles of the brain, is a circumstance of almost uniform occurrence, is fully demonstrated by the testimony of Professor Chapman, Drs. Barkhausen, Howship, Blake, Snowden, Shirlitz, Hunt and Peace.

Eighthly. That there is, in some instances, a peculiar emptiness of the vessel of the brain, or a pale, watery blood, in the large veins of the pia-mater, we are authorized to conclude, from the statements of Drs. Barkhausen, Sutleffe, and Mr. Howship.

*Stomach.*—As this is the organ upon which the morbific impression is made distinct, traces of abnormal action should be discovered. Thus we are informed by Professor Chapman, that he has uniformly found this organ in a state of high inflammation. In two instances, it is remarked by Dr. Snowden, that the stomach "was florid, and appeared to have been highly inflamed." By Barkhausen we are told, that the mucous membrane of the stomach was found reddened. The same observation is made by Dr. Lind. We are told by Dr. Peace, that "some few red specks existed," some of the vessels of the stomach "were distended, and exhibited an arborescent arrangement." We are not advised if these morbid appearances were considered as the result of inflammation. In the case, however, of Ellen Mooney, we are told by Dr. Peace, that there were "patches of inflammation at the large extremity of the stomach." Although it may be considered exceptionable, we cannot refrain from adducing the evidence of Dr. Staughton, in illustration of the point under consideration. This physician says, that the dissections which he has made himself, or in which he has participated, uniformly disclosed an inflamed stomach. By Dr. Coates, inflammation of this organ is altogether denied. This, to a certain extent, is doubtless true, for there are unquestionable instances of this disease, in which no trace of inflammatory action can be detected, In others, however, the authorities just appealed to satisfactorily demonstrate that inflammation does exist in the stomach, and sometimes in a most perfect form.

Why facts in illustration of the state of the stomach in delirium tremens, are not more numerous and diversified, is ascribable to two substantial causes. In the first place, for a considerable number of years, it was not suspected that the stomach was at all implicated in this disease. In consequence of this, post mortem examinations were not extended farther than to ascertain the condition of the brain. In the second place, it has frequently happened, that permission could not be obtained to examine more than the contents of the cranium.

*Liver.*—In those who have for a considerable length of time indulged in the practice of frequent intoxicasion, the liver is rarely, if ever, found in a perfectly sound condition. In a state of intense predisposition, it cannot avoid being deeply implicated in delirium tremens. Thus, in the dissections made by Drs. Armstrong and Brown, this organ was in a state of congestion. By Professor Chapman, we are told, that in his dissections, the liver has invariably appeared variously diseased, and among numerous proofs of chronic derangement, there were "sometimes recent inflammatory marks." It is stated by Dr. Snowden, that "this organ always exhibited appearances of a preternatural character." In two subjects who had experienced repeated attacks of this disease, on making an incision into the posterior part of the right lobe of the liver, my knife was followed, in one, by a *pint*, in the other, by nearly a *quart* of sanious pus." Dr. Barkhausen says, that "besides the alterations which have been described as occurring in the brain and membranes, morbid appearances were discovered also in other important organs, particularly in the liver and spleen, both of which are, it is well known, often the seats of disease in drunkards." In a dissection which was made by M. Maunoir, the liver was found very much enlarged; it was of a lighter hue than ordinary, and there were tubercles in its parenchyma in a state of suppuration. Proof to any extent of the existence of a chronic disease of this organ, might be easily adduced, but it is our wish to confine our remarks to those appearances that have resulted from recent abnormal action. That we have not been able to produce evidence of the implication of the liver in delirium tremens from a greater variety of sources, will not surprise the reader, when is he informed, that it has not engaged the particular attention of any author who has investigated the nature and seat of this disease. Indeed, it has been generally considered *hors de combat*, and what has been said of it has been more the rusult of accident than design.

*Anomalous morbid appearances.*—By Dr. Staughton, the bowels and peritoneum were found inflamed. The colon was seen by Dr. Peace of an uniform redness; the lining membrane of the aorta and pulmonary artery, the valves, particularly the semilunar, of this artery, and the mitral of the left ventricle, were seen of a reddish hue, by Dr. Washington; and Dr. Snowden has seen the pericardium distended, by a preternatural quantity of water.

In the preceding necroscopic investigation, several morbid appearances were noticed, which, it is manifest, are not necessarily a part of the disease under consideration. This remark is especially applicable to serous effusion and sanguineous extravasation, which are the incontestible effects of an action previously instituted in the brain. The congestion of the cerebral veins, which was noticed in several instances, evidently supervened, while the patients were in *articulo mortis*. This is a common case in almost every fatal malady. We have, therefore, no satisfactory reason to authorise the supposition, that the congestion of the veins of the encephalon in delirium tremens, is essentially unlike that which is of a very common occurrence in almost every affection, during the convulsive throes of disolution. In this organ there is conclusive evidence of vascular irritation, inflammation, and nervous irritation. These are the only marks of disease which it can be pretended are primitive.

In the stomach, it is impossible to deny that, in some instances, there are marks of irritation and inflammation; and that in others, the evidence of inflammatory action is entirely absent. The same declaration is true of the liver, and doubtless had that organ been more closely attended to, the evidence of its participation in delirium tremens would have been more diversified and conclusive. Any traces of abnormal action in other tissues, is unworthy of particular notice. We shall now attempt to determine the organ primitively affected in this disease."—pp. 49--53.

Our author now proceeds to consider the proximate cause of Delirium Tremens, and particularly to determine the organ primarily affected. He proposes to show that this cause

"Consists originally either in vascular irritation, actual inflammation, or nervous irritation of the stomach, with immediate sympathetic vascular irritation, actual inflammation, or nervous irritation of the liver, and, subsequently, of the brain."—p. 53.

We have not space to introduce a statement of our author's reasonings in support of this proposition. They run through more than 25 pages; and are chiefly devoted to the support of the gastric origin of Delirium Tremens as asserted by Dr. Klapp, and to an examination of Dr. Coates' objections to that pathology. Dr. Cross does not, however, believe that gastritis is always present, though the first link of the morbid chain is in the stomach: it may be inflamed or only irritated. In the course of this discussion, Dr. Cross has introduced from various sources a number of interesting facts relative to the influence of disease of the chylopoietic viscera, in the production of mental alienation, which are worthy of the attention of all who would comprehend the production of the latter. Referring to the work itself for a full account of his pathological speculations, we shall proceed to give some account of the treatment proposed.

"By Dr. Pearson it is asserted, that if the lancet is used, "the odds against the patient's existence, let him be ever so robust, are at least twenty to one." By Dr. M'Whirter it is said, that he has been "well assured, that when blood-letting, blistering, and abstinence have been prescribed, the patients have to a man died." It is said that three cases treated by Mr. Hunt, on the antiphlogistic plan, terminated fatally. Dr. Stewart, who, we are informed, has seen much of this disease, enters his protest against blood-letting. It is proscribed by Dr. Johnson. By Dr. Ayre, this disease is treated exclusively upon the narcotico-stimulant plan. Without hesitation it is declared by Dr. Coates, that the lancet "does not appear to have the least discoverable effect, positive or negative, upon the delirium; and that it actually does harm to the patient, by diminishing his strength, an item highly necessary to the cure."

Dr. Snowden says, it may be necessary to take a few ounces of blood if the pain in the head be very intense. It is remarked by Dr. Sutton, that "at a very early stage of the paroxysm, if in a plethoric subject, blood may be drawn." By Dr. Stephen Brown it is alledged, that the use of this remedy "should be restricted to the early stage of the affection, and to habits where the stamina are not impaired by a long practice of intemperance." Of the same import is the advice given by Dr. Barkhausen. On this subject, it is stated by Dr. Armstrong, who is a host himself, that "in constitutions that have not been shaken by reiterated drunkenness, I have known early and moderate venesection of much use, especially when followed by active aperients."

Prof. Potter, whose high pretensions as a sound and successful practitioner have never been questioned, declares, that "in young subjects, and even in patients advanced in life, but recently attacked, we have frequently bled to the amount of seventy or eighty ounces, and several times an hundred in three or four days." In some instances so energetic are the symptoms indicative of inflammatory action, that Prof. Frank has been induced to conclude, that this disease is a peculiar *encephalitis*, and consequently in accordance with this view of the subject, the treatment is conducted exclusively upon antiphlogistic principles.

The position, that there are cases, which imperiously require the lancet, and others, in which its effects will be very equivocal, and others again, in which it will be positively deleterious, is absolutely incontrovertible.

The necessity for either general or local blood-letting does not frequently occur in *sthenic delirium tremens*. To the author, there has not yet occurred an instance, in which he considered the detraction of blood from the arm as authorised. That such a case might occur we can conceive, but I imagine, that it would more properly come under the head of hypersthentic than of this variety of delirium tremens. When a loss of blood is indicated we have always preferred the application of cups to the back of the neck or to the temples.

In *hypersthenic delirium tremens*, the indication is much plainer, and the propriety of the general detraction of blood is frequently imperiously necessary. Cases of this variety, are comparatively of rare occurrence, but when they do happen, and exhibit a certain degree of violence, as well might we attempt to cure it without the lancet, as common phrenitis. Among the earliest phenomena, convulsions and an apoplectic state of the brain, are sometimes found. Under such circumstances, so necessary is the lancet, that it needs no comment." —pp. 82--7.

In asthenic and bilious Delirium Tremens, particularly the former, the lancet in the opinion of our author, is contra-indicated. For ourselves we must say that we have seen but little benefit from the lancet in any case of Delirium Tremens, that has yet come before us.

*Emetics.* Dr. Cross discusses the value of emetics, as well as of blood-letting, by a reference to practical writers, the majority of whom he finds to be their eulogists. Notwithstanding this, he is persuaded that they do not possess any superior advantages, and he believes them sometimes injurious. The Dr. has come to the conclusion that when they do good, it is by acting on the liver and through it, on the brain, an explanation which we think quite gratuitous. That they exert a beneficial influence on the liver when disordered, we do not deny, but have as little doubt, that they exert a salutary influence on the brain independently of the liver. We think more favorably of their effects in Delirium Tremens than our author, though we certainly have not realized from them as many advantages as Dr. Klapp the American author of the practice. Dr. Cross advises against allowing the emetic medicine to pass off by the bowels, which is good counsel. To this end it is proper, where such an event can be anticipated, to administer laudanum in connection with the emetic solution, or to give pills of opium and tartarized antimony. The tendency of ipicac to act on the bowels is so much less than that of tartar emetic, as to constitute a recommendation in these cases, was it not that its emetic qualities are too feeble. Our author thinks that emetics are absolutely contra-indicated in hypersthenic Delirium Tremens; but this denunciation is far too sweeping.

*Very* acute inflammation of the brain accompanied with *great* power of the heart, is not likely to exist in this disease, for its remote cause must have enfeebled the circulation, before it could have so disordered the nervous function, as to induce this peculiar form of delirium. In its most acute varieties, then, after copious blood-letting, antimonial emetics may in our opinion be given with safety; especially if administered in small and repeated doses so as to excite protracted nausea. In these cases, should they act on the bowels, it will do no harm.

Dr. Cross is of opinion, that in the asthenic variety, emetics are "strongly indicated and particularly useful." When, however, the constitution is greatly impaired, and the circulation very languid, emetics may do harm by their prostrating influence. In such cases, the vomit should be dissolved in a strong infusion of valerian, (*valeriana sylvestris, radix;*) or instead of the Antimony, mustard should be used, as in the more advanced stages of Cholera, to which, in fact, asthenic Delirium Tremens has much physical assimilation. In cases of this kind, applications to the skin, as a hot and stimulating foot bath, and a sinapism to the epigastrium, are highly beneficial during the operation of the vomit. We entirely concur with our author in the favorable opinion he expresses of emetics where there is a bilious complication.

*Cathartics.* Dr. Cross speaks more favorably of Cathartics than most writers. He has never seen a case of the disease in its first stage, in which a Cathartic of Calomel, Rhubarb and Aloes was not positively indicated. These articles he prefers to all others. The saline and hydrogagne he condemns. In hypersthenic Delirium, he considers purging of great value: in the bilious variety it is equally called for. On the whole, his partialities for the Cathartic treatment are greater than our own, but we admit that in many cases it may be employed with great advantage.

"When bleeding or puking is indicated, the lancet and emetics should precede cathartics. By observing this precept, the action of the latter will be promoted; and, moreover, they will not then interfere with the operation of each other. This is a circumstance of considerable importance, and should not be neglected. Hurry and precipitation in the exhibition of such active remedies, might produce immediate exhaustion in a system the most robust. We have uniformly permitted the immediate effects of venesection to subside before a cathartic is exhibited, and where emesis is indicated, we suffer the stomach to become tranquil before purgatives are given."—p. 101.

*Opium.* Admitting that of all the remedies for Delirium Tremens, opium has been most extravagantly commended, our author regards it as worth while to ascertain the principles which should regulate its employment. We shall make from this part of his book the following extract.

"As a rule of general application, opium is never to be used except when the pulse is soft and compressible. To accomplish this, either local or general blood-letting is frequently necessary. The propriety, also of premising the use of this drug, even in cases where a pulse of this character exists from the beginning, by the exhibition of a cathartic, has been fully verified by the clinical observation of the author. Although we may be inclined to attach more importance to this measure, and enjoin its necessity more earnestly than most physicians, yet there are those who speak of its utility in particular instances, in terms of decided approbation. By Dr. Armstrong, if the patient be tolerably robust, the liberal use of purgatives is recommended to be continued through the first two or three days. It is said by Professor Chapman, that "after copious evacuations of the stomach, in which the bowels generally share, we may recur with the happiest effects to the opium and brandy treatment." By such authorities the correctness of any practice may be established.

The propriety of giving calomel in conjunction with opium, has been strongly urged by Dr. Armstong. His practice has, however, been condemned by Dr. Brown; but his objections spring exclusively from a contracted and erroneous view of its pathology. A review of the evidence in the preceding pages must convince the reader, that the liver is deeply implicated in delirium tremens, and that consequently, the interposition of calomel is necessary for its removal. Repeated experience has convinced us, that no article can be associated with opium, which will enable it to fill as many important indications as calomel. It stimulates the liver, and produces a prompt and powerful disgorgement of that organ; with opium, calomel has been demonstrated to possess a peculiar and prodigious power in the subjugation of vascular irritation and inflammation of the stomach; and to arrest vomiting, a symptom sometimes very annoying, calomel has not a superior. These are incontestible attributes of calomel, and it must, therefore, be signally serviceable in this disease.

The blue mass has been recommended in conjunction with opium. Of its efficacy, in this complaint, we have no knowledge. The tardiness, however, of its operation is an insuperable objection to it in the estimation of the author. Nor have we any personal acquaintance with the remediate virtues of the spirit, terebrenthinae when exhibited in consociation with opium in awakening the dormant sensibility of the stomach. To promote the operation of this drug, camphor has been associated with it. Of its powers in this respect we cannot speak with much confidence: but to prevent calomel from griping we have frequently used it, and very advantageously. Dr. Armstrong is of opinion that the efficacy of laudanum is considerably improved by its combination with æther. Of this we shall speak in another place.

In no disease, with the exception of *tetanus*, has opium been given in such enormous doses as in delirium tremens. As much as one hundred grains have been exhibited and with complete success. This undaunted enforcement of opium is sometimes unavoidable, but we are, nevertheless, convinced, that a more prodigal use is commonly made of this powerful narcotic than is absolutely necessary. Much of this enormous expenditure may be curtailed by properly preparing the system for its administration, and by its association with suitable auxiliaries. These are circumstances which deserve the closest attention. By judicious management they may be made eminently subsidiary to success.

Let us not, however, be understood to mean, that this will always enable us to subdue delirium tremens by what may be considered a moderate quantity of opium. If such an impression is made, the physician will be disappointed. Rebellious cases will occasionally occur, notwithstanding every preparatory precaution may have been taken, in which it will be imperiously necessary boldly to enforce the exhibition of this remedy to the utmost possible extent. Under such ciscumstances, there is no point at which we can venture to say the physician should stop. The emergency of the case should be his only guide. After he has come to the conclusion that opium is the proper remedy, quantity should not deter him. Think not of its poisonous effects: subdue the disease at the hazard of poisoning the patient. Infinitely more safe is it to run the risk of bringing the system under the poisonous influence of opium, than not succeeding in completely subjugating the irritation of the stomach. Unrestrained in its action, the latter will surely extinguish life, but under proper management the former never will.

We speak thus confidently, because of the unlimited faith we have in the efficacy of cold affusions as a remedy for poisoning by opium. Experiment and experience have both assured us, that our confidence is not unfounded. In the hands of the author, though his trials with it have been considerably diversified, it has never failed. In spasmodic affections we have frequently had recourse to quantities of opium, upon which we never would have ventured but for the firm conviction, that our patients were always safe under the influence of cold water. On one occasion, we gave more than one hundred grains of solid opium in less than ten hours: the prostration was overwhelming, but without the aid of any auxiliary, the rigid enforcement of cold affusions at last proved completely triumphant.

We, therefore, assert, that after the system is properly prepared, and the necessity for its exhibition manifestly appears, no quantity should deter us. There is no reason for apprehension. To this declaration we are in part encouraged by the fact, that cold water itself has been warmly commended in the disease under consideration. This circumstance may, therefore, embolden us to have recourse to cold affusions with more freedom, as we are on high authority assured, that the interests of our patients will not be necessarily compromised."—pp. 106–9.

On the predisposing and associate measures, calculated to give greater efficacy to opium, than if that drug were given at once and alone; our author has a judicious summary of observations, some of which we shall extract.

"In sthenic delirium tremens, after the bowels have been freely opened and the liver stimulated to the production of a copious discharge of bile by the action of a dose of calomel, aloes and rhubarb, if the pulse should be soft and compressible, we commence the exhibition of calomel and opium in conjunction. Twenty grains of the former and three of the latter is the dose we ordinarily administer in the commencement. In three hours we repeat the opium alone, and in three hours more the same quantity of calomel and opium is repeated. In two or three hours after the exhibition of the last dose, fifteen grains of aloes and the same quantity of rhubarb is given. This simple plan has never failed of success but in one single instance. Indeed, it sometimes happens that the patient is sleeping soundly before the time arrived for the exhibition of the aloetic cathartic. When, however, sleep does not occur after the operation of the cathartic, as happened in the failure just alluded to, the same course is to be repeated, but more energetically, and the disease will unquestionably yield.

By this system of treatment the bowels are relaxed and the liver disgored; circulation is equalized, and vascular irritation of the stomach is promptly subdued. Several beneficial purposes are answered by the exhibition of a cathartic after we suppose we have given opium and calomel enough to remove the disease. Before the time has arrived for the administration of aloes and rhubarb, the opium and calomel will have accomplished all that we expect from them, but as it is possible this course may have to be repeated, it is important that the bowels should be evacuated to prevent their accumulation. Moreover the calomel retained by the opium may excite salivation. Although this is not a circumstance of much consequence, it should be avoided when possible. The liver under the action of mercury pours out bile exuberantly, and as it is sometimes acid, it may prove a source of considerable irritation, and thus defer and even prevent sleep after the disordered state of the stomach has been entirely removed.

In hypersthenic delirium tremens, preliminary to the use of opium, the lancet and cathartics should be energetically enforced until the pulse becomes manifestly soft and compressible. In conjunction with the lancet, purgatives are the most powerful means of diminishing cerebral determination. It will be necessary, therefore, in a majority of cases, to use them freely during the first two or three days. But we shall be disappointed if we expect to subdue the symptomatic inflammation of the brain. All we can do is to moderate the symptoms so as to enable the opium and calomel to act efficiently. It is perfectly clear that the secondary affection cannot be removed before the primitive inflammation is subdued. To bleed and purge, therefore, until the inflammation of the stomach is completely subjugated, would be to force the system in a number of instances, into a state of irremediable prostration. We are well aware that this disease has yielded to a treatment purely antiphlogistic. This, however, we cannot but consider unnecessarily hazardous. If there was no other alternative, we should be obliged to have recourse to it, but as we have at command remedies not only more safe, but decidedly more effectual, we feel authorised in condemning a treatment altogether depletory.

The controlling influence exercised by opium and calomel over inflammatory affections of the mucous and serous membranes, has been incontestibly evinced. In behalf of these articles we have only to appeal to the testimony of Mr. Annesley and Dr. Armstrong. Numerous other sources of evidence might be laid under contribution, but these are sufficient. The efficacy of no treatment rests upon proof more diversified, concurrent and irrefragable. By the author, the effects of these articles in membriform inflammation have been closely studied for several years, and from an experience of their virtues in a great variety of cases, he declares as the deliberate conviction of his mind, that where judiciously managed, they are decidedly superior to the lancet itself.

When, therefore, we have subjugated the morbid energy of the vascular system, the indication of which is a soft and compressible pulse, to subdue gastric inflammation, we have immediate recourse to opium and calomel. Nor can we bring the system under their influence too expeditiously. They must be energetically enforced, and the readiness with which they will frequently remove gastric inflammation, will excite the wonder and astonishment of the practitioner. We pursue the same course here, so confidently recommended in the sthenic variety, except, that here the doses should be considerably larger. We rarely commence with less than four grains of opium and half a drachm of calomel. But in this variety, the disease will not as frequently yield to the first trial, as in the sthenic. I have not known it necessary to make more than one repetition in augmented doses. It is true, we have had but a very limited number of cases of the hypersthenic variety; but those that have fallen under our notice, have never required more than twenty-four grains of opium to accomplish their complete cure. But we have seen too little of this variety to assert confidently, that more may not in some cases be necessary. The quantity, however, under no circumstances, should be our guide: the effects produced are the only data from which sound inferences can be drawn.

In asthenic delirium tremens, as there is a feeble and very frequent pulse, physicians have had recourse to opium from the very beginning, and without making any attempt to prepare the system for its successful exhibition. This is a course of treatment deduced manifestly from an empirical view of the subject. I cannot discover the principles upon which it is founded. Besides a torpid condition of the liver, which unquestionably demands the most urgent attention, there is an obstinate state of constipation, which has arrested the attention of the most careless observers. To relax the bowels, is a preliminary step in the treatment of all the diseases with which we are acquainted, with the exception of delirium tremens, an affection which presents *prima facie* evidence of the necessity and importance of it. So plain an infraction of a fixed principle in clinical medicine, should not be committed, except for the most conclusive reasons."—pp. 113–16.

Of the great value of calomel and opium combined, in the treatment of sub-acute inflammations and in nervous irritations, we have more than once expressed ourselves in this Journal; and therefore on this occasion need only say that we concur in the foregoing observations.

In bilious Delirium Tremens, our author thinks there are two important indications;—to subdue vascular irritation, and prevent or extinguish nervous irritation.

"To remove vascular irritation, the lancet, emetics and cathartics, have been, under ordinary circumstances, the most effectual. In consequence of the enfeebled condition of the system, there is generally very inefficient excitement. This not only renders unnecessary, but absolutely precludes, the detraction of blood. This is a measure upon which we have never ventured. The prominency of the gastric symptoms readily reveals the necessity of emetics. In the exhibition of articles to produce this result, we are to be guided by the rules already delivered. But in the early stage of this variety, our reliance is upon calomel, aloes and rhubarb. The engorged condition of the liver is not only great, but manifest. If there is no tenseness or fulness of the pulse, it will not be safe to give more than one dose of these active medicines before we have recourse to such means as will have a tendency to alleviate nervous irritation. But in the event of the presence of such attributes of the pluse as have been just mentioned, we may safely and successfully keep the bowels and liver under the constant action of the articles above suggested, until it becomes soft and compressible. Although it may be necessary afterwards to purge, it would be rather hazardous to push cathartics farther at present, before, or unassociated with, the interposition of other articles of a very different tendency. If, however, the physician has availed himself of the period of time which frequently elapses before the occurrence of a compressible pulse, to purge actively and effectually, he may succeed in completely subduing reaction. If then he boldly and energetically exhibits the sulphate of quinine, or any other suitable tonic, he may in some cases prevent the developement of the characteristic symptoms of delirium tremens altogether. We must not, however, encourage the belief, that this is an event of frequent occurrence. In eight tenths of the cases of this variety, the constitution has been so extensively impaired by previous habits of intemperance, that the active operation of a single cathartic will produce a compressible pulse. After this every alvine evacuation that is produced, unless the system is at the same time under the influence of corroborant medicines, will exasperate nervous irritation, and hasten the developement of delirium.

While it is now manifest, that the interposition of some article that has a direct tendency to remove nervous irritation, is imperiously called for, it is equally true, that the secretion of bile must be free and unobstructed. It is utterly impossible to dispense with calomel. Some means must, therefore, be devised for its safe and sufficient exhibition. While the liver is exposed to its action, nervous irritation must be removed, and the strength of the system must be supported. To extinguish nervous irritation, the syrup of morphine has answered my warmest expectations; and, to support the strength of the system, no article could be better adapted than the sub-carbonate of ammonia." —pp. 121—2.

Our author enumerates camphor, henbane, belladonna, spider's web and hop, among the narcotics recommended for this disease, but finds no great amount of authority in their favor. Blisters to the head he thinks injurious, but has found a plaster to the pit of the stomach of much benefit.

*Cold Affusion.* Our author does not seem to value this very highly, but the authorities which he cites, are in its favor.

"When there is considerable determination to the head, indicated by much pain and heat, we may venture to apply cloths dipped in cold water to the temples and forehead, and with considerable advantage. Care must, however, always be taken not to continue them so long, or repeat them so often, as to produce much constitutional impression.

It may be proper to advertise the reader, that opium and cold affusions are incompatable remedies. This assertion we make upon the authority of experience, both ample and diversified. While under the influence of cold affusions the system can never be induced to come under the sedative control of opium, and when the latter has produced any impression, its force is completely neutralized by the administration to a sufficient extent of the former. When, therefore, the use of opium is indicated, we should be exceedingly careful not to administer cold affusions. This we consider a practical precept which is to be rigidly observed; and so frequently is opium imperiously required, that the cases, it is evident, in which cold affusions may be with propriety employed are few, and in them it is only admissible in particular circumstances."—p. 130.

Many cases it appears to us will admit of the cold-dash to the head in a much more decided degree than our author would tolerate; and we very much doubt the existence of that incompatibility between the use of opium and the cold affusion, on which he insists,—at least to the degree which he alledges.

*Ardent Spirits.*—Dr. Cross discusses the question whether ardent spirits are necessary in the treatment of Delirium Tremens, and comes to the correct conclusion that they are not. We have no doubt but they may be made sometimes to do good, but the end may be attained by other and less objectionable means.

*Moral Treatment.*—The observations of our author on the moral treatment of the deranged from drink, are judicious. He recommends a mild government, with as much liberty as may be consonant with the safety of the patient; he might have added with the security of those about him, for this should never be lost sight of in our plans of regimen.

Although the maniac from drink is generally timid and disposed to escape from the dangers that seem to surround him, he is far from being at all times harmless. Should his temperament of mind be quarrelsome, overbearing or ferocious, he may at any time commit violence on those about him. We have several times witnessed this; and the melancholy act of Birdsall, who in a fit of Delirium Tremens, killed his wife, in the neighbourhood of this city, must be within the recollection of the readers of this Journal, in which a medico-juridical narrative of his case was inserted.

As our author has attempted a monograph of this species of Delirium, it would have been proper for him to have considered it in a legal and moral view, which he has not done. For ourselves we do not hesitate to express the opinion, that the *non compos* whose mental alienation is denominated Delirium Tremens, or mania a potu, should be regarded as equally irresponsible to the law, with the *non compos* who is called a lunatic; but having discussed this subject, in previous numbers of the Journal, we shall not resume it in this.

Having given a tolerably full account of Dr. Cross' Prize Dissertation, we shall bring this article to a close, by saying that we regard it as a creditable production, comprising a much greater amount of matter on the subject of which it treats than any other essay that has fallen under our observation. Dr. Cross is one of our most studious and indefatigable young physicians; and his dissertation presents much correct thinking, and many favorable indications of good scholarship, but his style would be improved by using fewer words, and, by a simpler collocation.

